# Regulation of Kv2.1 biogenesis and gating by candidate disease-linked Kv6.1 variants

**DOI:** 10.1016/j.jbc.2025.110943

**Published:** 2025-11-13

**Authors:** Damayantee Das, Shawn M. Lamothe, Nicholas C. Duta, Chloe C. Koens, Yubin Hao, P.Y. Billie Au, Harley T. Kurata

**Affiliations:** 1 Department of Pharmacology, Alberta Diabetes Institute, University of Alberta, Edmonton, Alberta, Canada; 2 Department of Medical Genetics, Cumming School of Medicine, Alberta Children's Hospital Research Institute, University of Calgary, Calgary, Alberta, Canada

**Keywords:** anatomical dysmorphism, genetic disease, Kv2.1, Kv6.1, phosphorylation, posttranslational modification (PTM), potassium channel

## Abstract

Silent voltage-gated potassium channel subunits are enigmatic ion channels in the Kv5, Kv6, Kv8, and Kv9 families, which do not generate functional homomers, but instead assemble and modify the function of Kv2 family channels. While these biophysical consequences have been recognized, knowledge on physiological roles and pathologies associated with silent voltage-gated potassium channel subunits is not well developed. In this study, we investigated the functional effects of two Kv6.1 variants identified in either a pediatric patient (Kv6.1[L284P]), or a calf (Kv6.1[W416C]), which exhibited anatomical abnormalities. Kv2.1 current was reduced significantly when coexpressed with either Kv6.1 or Kv6.1[L284P], and even more dramatically with Kv6.1[W416C], which led to nearly complete suppression of Kv2.1 current. These mutants also attenuated Kv6.1-mediated effects on inactivation of Kv2.1. Interestingly, protein expression levels of Kv6.1 variants are not significantly affected when subunits are expressed alone. However, coexpression with Kv2.1 decreases expression of both Kv2.1 and Kv6.1 (and variants), and this effect is particularly powerful for Kv6.1[W416C]. Additionally, we report that Kv2.1 promotes phosphorylation of Kv6.1, but this is largely absent in the Kv6.1[W416C] mutant. Overall, effects of Kv6.1[W416C] are prominent, while Kv6.1[L284P]-mediated effects are much weaker and often difficult to distinguish from WT Kv6.1, and a direct causal link to anatomical abnormalities will require further investigation. Our findings highlight that Kv6.1 and variants influence expression and gating properties of Kv2.1, and also that coassembly with Kv2.1 leads to previously unrecognized posttranslational modifications of Kv6.1.

Kv2.1 is a prominent delayed rectifier potassium channel in the mammalian brain, present in most neuron types (1, 2, 3, 4, 5). These channels exhibit a wide range of potential functional diversity achieved through assembly with silent Kv (“KvS”) subunits (6, 7, 8, 9, 10, 11, 12, 13, 14, 15), association with auxiliary subunits (10, 16, 17), and posttranslational modifications (18, 19, 20, 21). Kv2 channels are generally thought to be unique in their assembly with the “KvS” family of silent subunits, encoded by the Kv5 (*KCNF*), Kv6 (*KCNG*), Kv8 (*KCNV*), and Kv9 (*KCNS*) gene families, although there is also a recent report of modulation of Kv7 channels by several different KvS subunits (22). The KvS subunits do not form functional homotetramers, despite possessing many hallmarks of a Kv channel, including an N-terminal T1 domain, voltage-sensing domain, and pore domain containing a canonical K^+^ selectivity filter signature sequence. Rather, the most widely recognized effects of KvS subunits are related to assembly with Kv2.1 and modulation of its biophysical properties (9). Depending on the KvS subunit coexpressed with Kv2.1, a variety of gating and trafficking effects may arise. For example, Kv6.1 coassembly with Kv2.1 is reported to decelerate the deactivation rate, promote a hyperpolarizing shift of voltage-dependent activation, and most prominently, shift the voltage dependence of inactivation to very negative voltages (8, 10, 23).

Kv2.1 mutations have been linked to a range of severe neurological outcomes including epileptic encephalopathy, developmental delay, intellectual disability, and autism (24, 25, 26, 27, 28), whereas Kv2.2 mutations have been recently linked to neurodevelopmental delay accompanied by anatomical abnormalities (29). However, less is known in terms of pathologies related to Kv6.1 or other KvS subunits. Mutations in the KvS subunit, Kv8.2 have been shown to influence mild febrile seizure and epileptic encephalopathy with severe refractory seizure (30, 31). Additionally, Kv6.4 is proposed to influence the development of migraine, as a Kv6.4 missense mutation (L360P) has been identified in migraine patients (32), and nearly completely suppresses functional Kv2.1 current (33).

In this study, we focused on the effects of Kv6.1 mutations on Kv2.1 function and expression, motivated by the observation of anatomical abnormalities (among other symptoms) identified in two different organisms carrying Kv6.1 mutations. We report identification of the L284P mutation in a pediatric patient with bilateral radial ray anomalies, alopecia, and small stature. The W416C mutation was previously described in a calf (bovine) with craniofacial dysmorphism accompanied by additional neuromuscular symptoms, although functional effects of this mutation were not characterized (34). We highlight mixed effects of L284P and especially W416C mutations in Kv6.1 on current density, inactivation, and expression of Kv2.1. We also focused on the interplay between Kv6.1 and Kv2.1 expression and posttranslational modifications, revealing features of Kv6.1 biogenesis that differ between WT Kv6.1 and W416C. Although still unclear given there is only a single human patient, these findings may indicate a previously unrecognized potential role of Kv6.1 in limb development and other anatomical deformities. There is a lack of strong evidence for Kv6.1 involvement in neurodevelopmental disorders, but its powerful modulation of Kv2.1 may also be important due to the identification of pathogenic variants of Kv2.1/*KCNB1* in developmental delay and epileptic encephalopathy (35, 36).

## Results

### Clinical presentation of a KCNG1 variant of unknown significance

**Figure 1:**
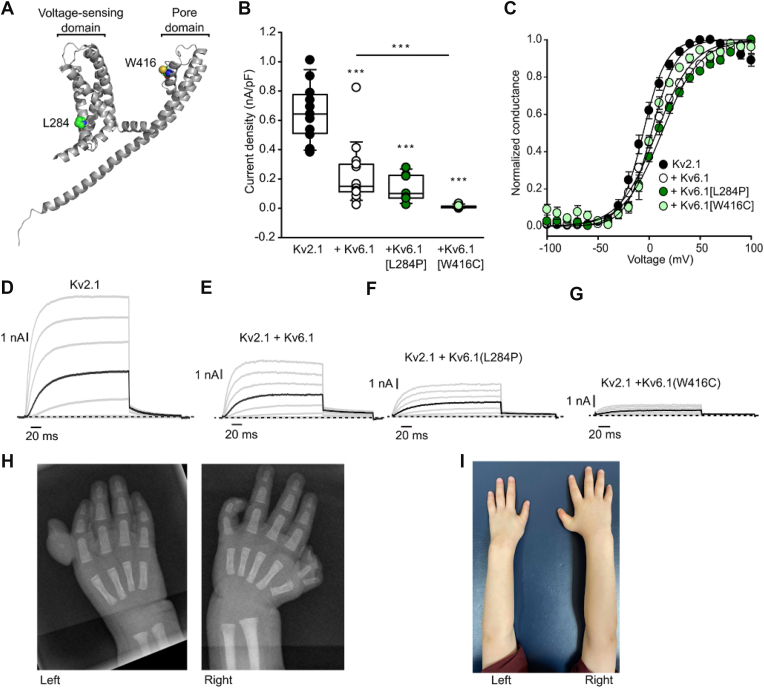
**Kv6.1 variant effects on Kv2.1 current magnitude and activation properties.***A*, AlphaFold structure of Kv6.1 depicting the position of the [W416C] mutation in the pore region and [L284P] mutation in the voltage sensing domain. *B*, current density at +30 mV was measured from indicated subunit combinations (1:1 cDNA ratio) after transient transfection in LM cells. One-way ANOVA followed by Tukey *post hoc* test was used to compare experimental groups, with ∗∗∗ indicating *p* < 0.001. *C*, conductance-voltage relationships for Kv2.1 coexpressed with each of the indicated Kv6.1 channels. Cells were stepped between −100 mV and +100 mV (200 ms in 10 mV steps, −100 mV holding potential), followed by a pulse to −30 mV for tail current measurement. Normalized tail current amplitudes were fit with Equation (1), (Kv2.1 V_1/2_ = −5.9 ± 2.5 mV, k = 11.5 ± 0.5 mV, n = 13; Kv2.1 + Kv6.1, V_1/2_ = 7.0 ± 2.1 mV, k = 18.5 ± 0.7 mV, n = 14; Kv2.1 + Kv6.1(L284P) V_1/2_ = 12.3 ± 2.0 mV, k = 19.0 ± 1.1 mV, n = 6; Kv2.1 + Kv6.1(W416C) V_1/2_ = 2.1 ± 2.4 mV, k = 12.3 ± 0.5 mV, n = 6). *D*–*G*, representative patch clamp records for Kv2.1 and indicated Kv6.1 variants. Voltage steps are shown in 20 mV intervals, and the darker sweep corresponds to +30 mV. The Kv2.1 control group was cotransfected with soluble GFP to ensure a constant amount of plasmid DNA for the transfection. *H*, X-rays of the L284P patient, with hypoplastic thumb in the *left* hand and duplication of distal and proximal phalanges in the *right* hand thumb. *I*, shortened *left* forearm in the same patient where the distal duplicated phalanx has been surgically removed and *left* hand is shown postpollicization.

Our investigation into *KCNG1*/Kv6.1 was prompted by the identification of a variant in *KCNG1* in a pediatric patient. The proband was a 2 year old boy seen in the Clinical Genetics Clinic (University of Calgary) for limb anomalies. He had a duplicated right thumb with a shortened left forearm (Fig. 1, *H* and *I*) and was also born with a left dangling thumb (Fig. 1*H*). He had two patches of congenital alopecia, unilateral cryptorchidism, and smaller stature, with height and weight tracking between the 3rd and 10th percentiles. Surgical intervention reoriented one of the digits of the left hand (Fig. 1*I*). Developmental milestones have been normal. He walked at 14 months and had 2 word phrases and short sentences by 2 years of age.

Chromosome microarray testing identified a deletion of 16q24.3 overlapping the first intron of *ANKRD11* that was inherited from an unaffected father who has postgraduate university level education. Clinical exome sequencing was then organized as a trio based test through Blueprint Genetics (initially in 2021 and with reanalysis in 2024) with both parents and the proband sequenced, which identified a *de novo* variant in *KCNG1* (NM_002237.4) c.851T>C, p.(Leu284Pro), which was of uncertain significance. There are currently six instances of this variant that appear in the gnomAD browser (allele frequency of 0.0000038). No other variants were identified.

### Kv6.1 and variants diminish Kv2.1 current

Kv6.1 is a “silent” modulatory Kv α-subunit, unable to form conductive channels on its own. However, Kv6.1 forms heterotetrameric channels by coassembling with subunits in the Kv2 subfamily, leading to altered gating properties including prominent effects on the kinetics and voltage dependence of deactivation and inactivation (8, 10). Based on the identification of the Kv6.1[L284P] mutation described above, we sought to compare Kv2.1 modulation by WT Kv6.1 *versus* Kv6.1[L284P]. We investigated an additional mutation (Kv6.1[W416C]), reported in a calf with developmental anomalies but not functionally characterized (34). The location of these mutations in the predicted structure of Kv6.1 (AlphaFold structure AF-Q9UIX4-F1) is highlighted in Figure 1*A*, with L284P lying in the voltage-sensing domain, and W416C within the selectivity filter region. At present, there is a lack of consensus regarding the stoichiometry of assembly of Kv2.1 and KvS subunits, thus a variety of stoichiometries of the mutant Kv6.1 subunits could potentially assemble with Kv2 subunits (37, 38).

We assessed currents generated by WT Kv2.1 coexpressed with Kv6.1 and each mutant (Fig. 1*B*; complementary DNAs (cDNAs) were transfected in a 1:1 ratio, and cotransfection with soluble GFP was used for Kv2.1 alone to ensure a constant amount of plasmid DNA in the transfection mixture), using patch clamp electrophysiology. These experiments revealed a significant reduction of Kv2.1 current density when coexpressed with WT Kv6.1 (Fig. 1*B*). This reduction in current was not explicitly reported in early studies of Kv6.1, although coexpression with the closely related Kv6.4 subunit also causes reduced current density at similar transfection ratios (37). Current suppression was even more exaggerated after coexpression of the Kv6.1[W416C] mutant, which nearly completely abolished currents, and a significant difference was observed between current density in Kv2.1+Kv6.1 *versus* Kv2.1+W416C groups (Fig. 1*B*). Although there were prominent effects on current magnitude, Kv6.1 and the mutant channels only caused modest shifts of the voltage dependence of activation (Fig. 1*C*). Exemplar traces of each group include sweeps to +30 mV that have been highlighted (bold black) for comparison (Fig. 1, *D*–*G*). These effects of Kv6.1 coexpression vary slightly from the small hyperpolarizing shift that was previously reported for Kv2.1+Kv6.1 channels (8, 10), but overall the effects on activation do not appear to be markedly altered by the mutant forms of Kv6.1, and were not investigated any further.

### Kv6.1 mutants attenuate effects on Kv2.1 inactivation

**Figure 2:**
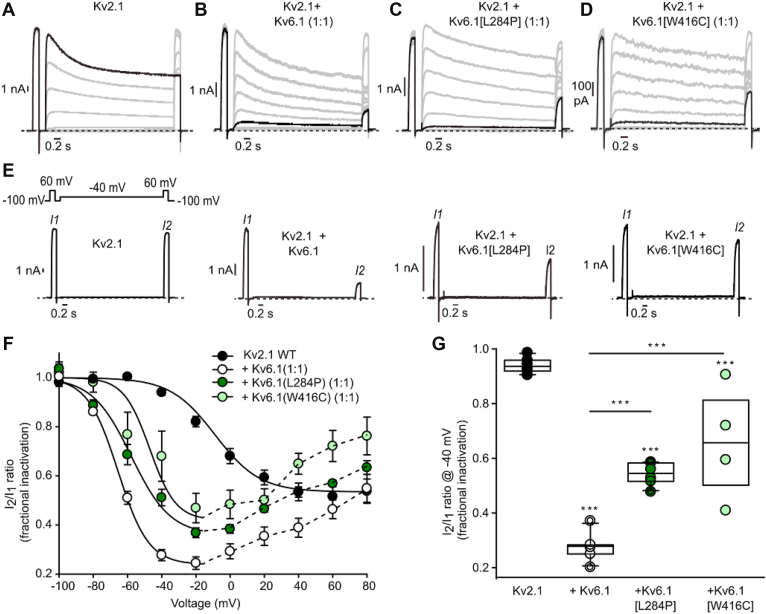
**Modulation of Kv2.1 inactivation properties by Kv6.1.***A*–*D*, exemplar recordings of indicated combinations of Kv2.1 and Kv6.1. To assess inactivation, cells were pulsed to 60 mV for 200 ms (I_1_) repolarized briefly to −100 mV, then stepped between −100 mV to +80 mV in 20 mV increments (4 s conditioning pulse) to induce channel inactivation, followed by another 200 ms test pulse to +60 mV (I_2_). The ratio of I2/I1 reflects the extent of inactivation during the conditioning pulse. Sweeps leading to maximal inactivation are highlighted in *black* each panel (+60 mV for Kv2.1, −20 mV Kv2.1 + Kv6.1 and variants). The Kv2.1 control group was cotransfected with soluble GFP to ensure a constant amount of plasmid DNA in the transfection. *E*, single sweeps from the inactivation protocol, with a conditioning step to −40 mV to illustrate inactivation at intermediate voltages in each group. *F*, mean voltage dependence of inactivation, determined by the ratio of I2/I1 in each sweep (n = 4–8 per group). *G*, fractional inactivation (measured using I2/I1 ratio) at −40 mV was measured from indicated subunit combinations (1:1 cDNA ratio) after transient transfection in LM cells. One-way ANOVA followed by Tukey *post hoc* test was used to compare experimental groups, with ∗∗∗ indicating *p* < 0.001.

Kv2.1 exhibits slow inactivation upon sustained depolarizations (Fig. 2, *A*–*D*). We analyzed the voltage dependence of inactivation using a three-pulse protocol, in which the extent of inactivation was determined from the ratio of current sizes in pulses to +60 mV immediately before (I_1_) and after (I_2_) a series of 4 s conditioning pulses to voltages between −100 mV and +80 mV (Fig. 2, *A*–*D* and *F*). Note that currents have been scaled to emphasize the altered kinetics of inactivation in different Kv6.1 variants, but that the Kv2.1:Kv6.1[W416C] currents are particularly small, as reported in Figure 1. Thus, it was rare to obtain a cell with sufficiently large currents to generate a reliable inactivation-voltage relationship, and it should be noted that there is a possibility that homomeric Kv2.1 channels contribute to the currents and apparently weaker inactivation in the Kv2.1+Kv6.1[W416C] condition. Sweeps highlighted in black indicate the voltage where maximal inactivation was observed (+60 mV for Kv2.1, −20 mV for Kv2.1 coexpressed with Kv6.1 or variants) (Fig. 2, *A*–*D*). Interestingly, the voltage dependence of Kv2.1 inactivation varies somewhat in different expression systems. Some studies have reported a U-shaped voltage dependence of inactivation (23, 39), although our studies using LM cells (Mouse L(tk-) fibroblast cell line) result in less pronounced U-shaped voltage dependence, consistent with other recent studies using this expression system (Fig. 2, *A* and *F*) (38). In the context of Kv6.1 modulation, coexpression of Kv2.1 with Kv6.1 leads to markedly enhanced inactivation at hyperpolarized voltages, reflected in a shift of the steady-state inactivation curve, and deeper inactivation at intermediate voltages (Fig. 2, *A*, *B*, *F* and *G*) (7, 10), resembling the effects of coexpression with Kv6.4 (37, 38). In the exemplar currents in Figure 2*E*, the extent of inactivation during a 4 s pulse to −40 mV is highlighted, illustrating that Kv2.1 exhibits minimal inactivation (I_2_ ∼ = I_1_), whereas coexpression with Kv6.1 leads to prominent inactivation at the same voltage (reflected in the reduced size of current in the I_2_ pulse relative to I_1_). Coexpression with the Kv6.1 mutants also enhanced inactivation at intermediate voltages. However, there were graded effects in terms of weakened extent of inactivation in the Kv6.1 variants (Fig. 2, *C*, *D*, *F* and *G*). The Kv6.1 variants caused less complete inactivation compared to WT Kv6.1, throughout the voltage range tested. We compared the extent of inactivation at −40 mV, in the range of maximal inactivation, and observed a significant difference between the L284P and W416C variants relative to WT Kv6.1 (Fig. 2*G*). This is the most prominent gating disruption that we observed for the tested Kv6.1 variants.

### Reciprocal interactions underlie suppression of Kv2.1 by Kv6.1[W416C]

**Figure 3:**
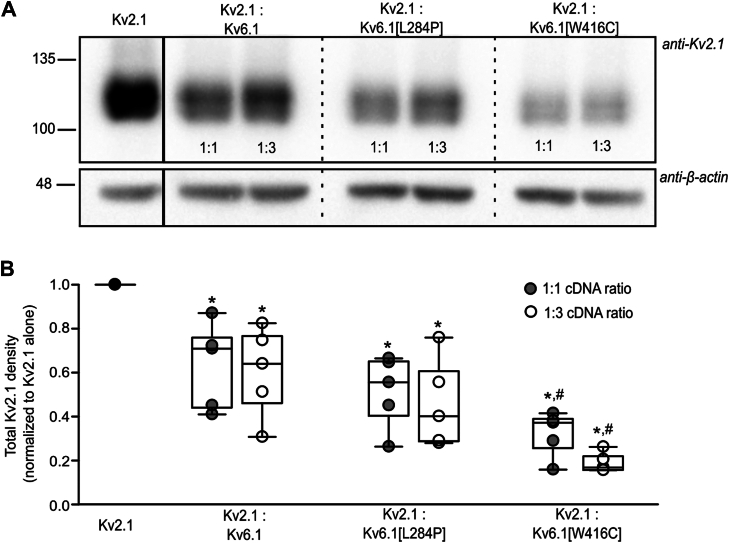
**Kv6.1 and variants influence total Kv2.1 expression.***A*, Kv2.1 was coexpressed with Kv6.1, Kv6.1[L284P], and Kv6.1[W416C] in 1:1 and 1:3 ratios as indicated, for 72 h in HEK293 cells. The Kv2.1 control group was cotransfected with soluble GFP to ensure a constant amount of plasmid DNA in the transfection. Whole-cell lysates were separated using SDS-PAGE and probed with anti-Kv2.1 and β-actin (loading control) antibodies. All the lanes in the representative blot are from the same blot. *B*, total expression of Kv2.1 in each group was first normalized to their respective β-actin signal and plotted as normalized to the Kv2.1 control expression in each experiment (n = 5). A repeated measures ANOVA test (on raw density normalized to β-actin) followed by a *post hoc* paired *t* test was used to compare experimental groups where appropriate (∗ indicates *p* < 0.05 relative to Kv2.1 alone and # indicates *p* < 0.05 relative to the Kv2.1:Kv6.1 condition).

We used Western blots to investigate expression of Kv2.1 with Kv6.1 and its variants (Fig. 3). Consistent with the reduced current density observed in Figures 1 and 2, coexpression of Kv2.1 with WT Kv6.1 or either mutant, results in reduced expression relative to Kv2.1 alone (Fig. 3*B*). Suppression of Kv2.1 expression was most prominent when coexpressed with Kv6.1[W416C]. Coexpression with Kv6.1[L284P] caused modest suppression of Kv2.1 expression that was not statistically different from WT Kv6.1, and the extent of suppression was variable. It is also noteworthy that Kv2.1 migrates as multiple bands on SDS-PAGE, previously attributed to varying degrees of phosphorylation of the channel (21). When coexpressed with Kv6.1[W416C], lower molecular weight forms of Kv2.1 are more prominent relative to the total signal (Fig. 3*A*), likely indicating a reduced extent of channel phosphorylation.

**Figure 4:**
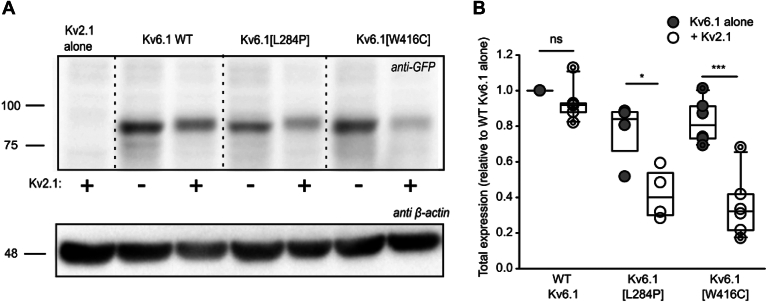
**Kv6.1 expression is diminished by coexpression with Kv2.1.***A*, Kv6.1 and variants were coexpressed with or without Kv2.1 in a 1:1 ratio as indicated, for 72 h in HEK293 cells. The Kv6.1 and variant control groups were cotransfected with GFP to ensure a constant amount of plasmid DNA in the transfection. Whole-cell lysates were separated by SDS-PAGE and probed using an anti-GFP antibody (Kv6.1 constructs are EGFP-tagged), using β-actin as a loading control. Protein ladder molecular weights are in kDa. *B*, total expression of Kv6.1 in each group was normalized to the β-actin loading control and is plotted as normalized to the WT Kv6.1 control (expressed alone) expression in the corresponding experiment (n = 4–6). A paired *t* test was used to compare the expression between experimental groups for Kv6.1 or each variant (∗ and ∗∗∗ indicate *p* values of *p* < 0.05 and *p* < 0.001, respectively).

In parallel, we assessed expression of WT Kv6.1 and the variant subunits. We used an EGFP-tagged Kv6.1 construct (N-terminal tag) for detection with an anti-GFP antibody, because commercially available Kv6.1 antibodies did not work consistently in our experiments (Fig. 4). Interestingly, while the Kv6.1 variants influence expression of Kv2.1:Kv6.1 heteromeric channels, effects on Kv6.1 expression alone are not as pronounced (Fig. 4). When expressed alone (*i.e.*, without Kv2.1), Kv6.1, Kv6.1[L284P], and Kv6.1[W416C] exhibit comparable expression levels. However, they respond very differently when coexpressed with Kv2.1. We observed a variable but generally modest decrease of Kv6.1 expression when coexpressed with Kv2.1, a similarly slight decrease of Kv6.1[L284P], whereas Kv6.1[W416C] was prominently suppressed when coexpressed with Kv2.1 (Fig. 4, *A* and *B*). This finding was also consistent with the effects on current density reported in Figure 1. These findings indicate that the Kv6.1 variants do not themselves weaken protein expression, but rather require coassembly with Kv2.1 in order to cause reduced protein expression.

In addition to these effects on overall expression, a prominent effect of Kv2.1 coexpression also emerges in WT Kv6.1 and Kv6.1[L284P] channels, in terms of their mobility on SDS-PAGE. Coexpression with Kv2.1 shifts the molecular weight of Kv6.1 and Kv6.1[L284P] slightly higher, suggesting assembly-dependent posttranslational modification of Kv6.1 (*i.e.*, modifications that only occur when Kv6.1 is coassembled with Kv2.1). This did not appear to occur with Kv6.1[W416C] although the signal intensity on Western blots was generally too low to be absolutely certain. The origins of this band shift are discussed later.

### Coassembly of Kv2.1 and Kv6.1

**Figure 5:**
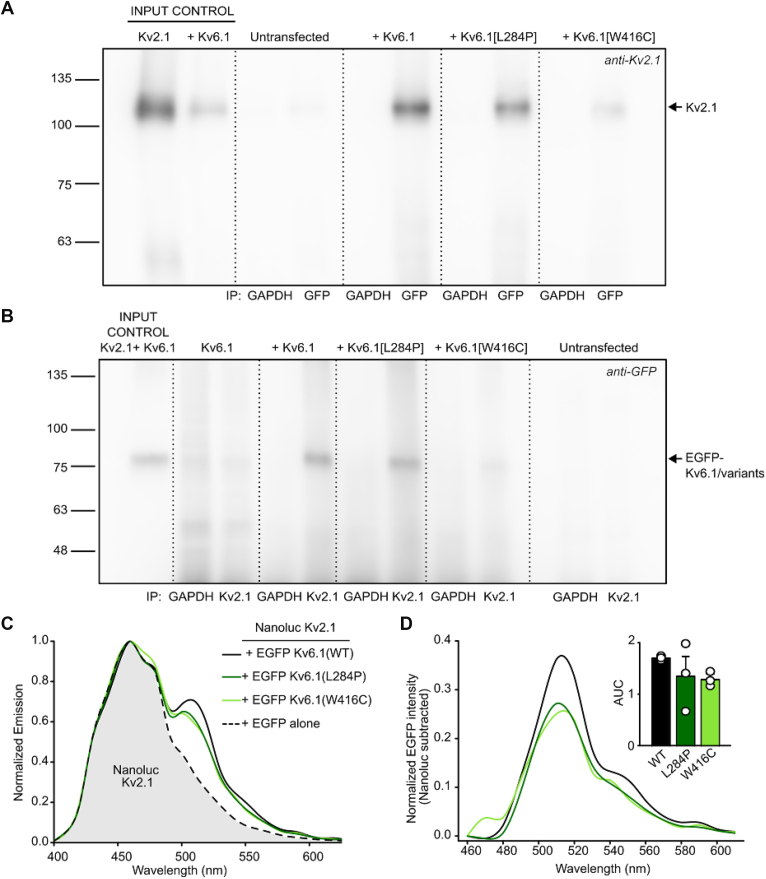
**Kv2.1 association with Kv6.1 and variants.***A*, coimmunoprecipitation of Kv2.1 by EGFP-Kv6.1 variants. Cell lysates were incubated with either anti-GFP or anti-GAPDH (negative control) as pull-down antibodies, and immunoprecipitates were probed with anti-Kv2.1. *B*, conversely, pulldowns were done with anti-Kv2.1 or anti-GAPDH (negative control), and immunoprecipitates were probed with anti-GFP (n = 3). For both *panels A and B*, the “+ Kv6.1 or + variants” groups indicate “Kv2.1+ Kv6.1 or variants.” *C*, cells were transfected with Nanoluc-Kv2.1 and EGFP-Kv6.1 (WT + variants), as indicated, and emission spectra were collected during incubation with Nano-Glo (Promega) substrate. *D*, spectra for each Nanoluc-Kv2.1 + EGFP-Kv6.1 variant were subtracted from the Nanoluc emission spectrum, to illustrate the appearance of the EGFP BRET acceptor signal (n = 3). The inset bar graph summarizes the area under the curve (AUC) of the EGFP signal for each condition. The control group (Nanoluc-Kv2.1 alone) was co-transfected with soluble GFP to ensure a constant amount of plasmid DNA in the transfection mixture for all experiments. BRET, bioluminescence resonance energy transfer.

It is well accepted that Kv2 subunits directly assemble with KvS subunits in cell lines and *in vivo* including Kv5.1 (40, 41). In addition to our functional investigation of Kv2.1 modulation by Kv6.1, we investigated physical coassembly of these full-length proteins and whether this was affected by Kv6.1 variants. We used coimmunoprecipitation to demonstrate that Kv2.1 and Kv6.1 (and variants) are closely associated, consistent with formation of heterotetrameric channels (Fig. 5). In this assay, we found that anti-GFP (directed toward EGFP-Kv6.1) immunoprecipitates consistently pulled down Kv2.1 (*versus* anti-GAPDH negative control), and this was observed for each of the Kv6.1 variants (Fig. 5*A*). As expected, expression of Kv2.1 was strongly suppressed when coexpressed with Kv6.1[W416C]; however, there was still a clear detection of Kv2.1, indicating that the mutation does not disrupt association with Kv2.1 (Fig. 2*A*). We also demonstrated the interaction in reverse using an anti-Kv2.1 antibody pulldown (Fig. 5*B*), with clear identification of Kv6.1 and each of the two variants in the anti-Kv2.1 immunoprecipitate (again with weaker abundance of Kv6.1[W416C]).

We also assessed proximity of Kv2.1 and Kv6.1 variants using a bioluminescence resonance energy transfer (BRET) approach (Fig. 5*C*). NanoLuc-Kv2.1 (BRET donor) and EGFP-Kv6.1 (BRET acceptor) were co-expressed, followed by spectral analysis. Expression of NanoLuc-Kv2.1 alone leads to the appearance of a characteristic NanoLuc emission spectrum with an emission peak of ∼460 nm (Fig. 5*C*). Coexpression with EGFP-Kv6.1 or its variants leads to emergence of an additional emission peak near 515 nm (Fig. 5, *C* and *D*), arising from energy transfer between NanoLuc and EGFP. This indicates close association between Kv2.1 and each of the Kv6.1 variants. Please note that these fusion proteins were generated with tags on the N terminus of Kv2.1 and Kv6.1, and the interaction of their T1 domains likely contributes to positioning the BRET pair in close proximity.

### Microscopy-based assessment of Kv2.1 expression

**Figure 6:**
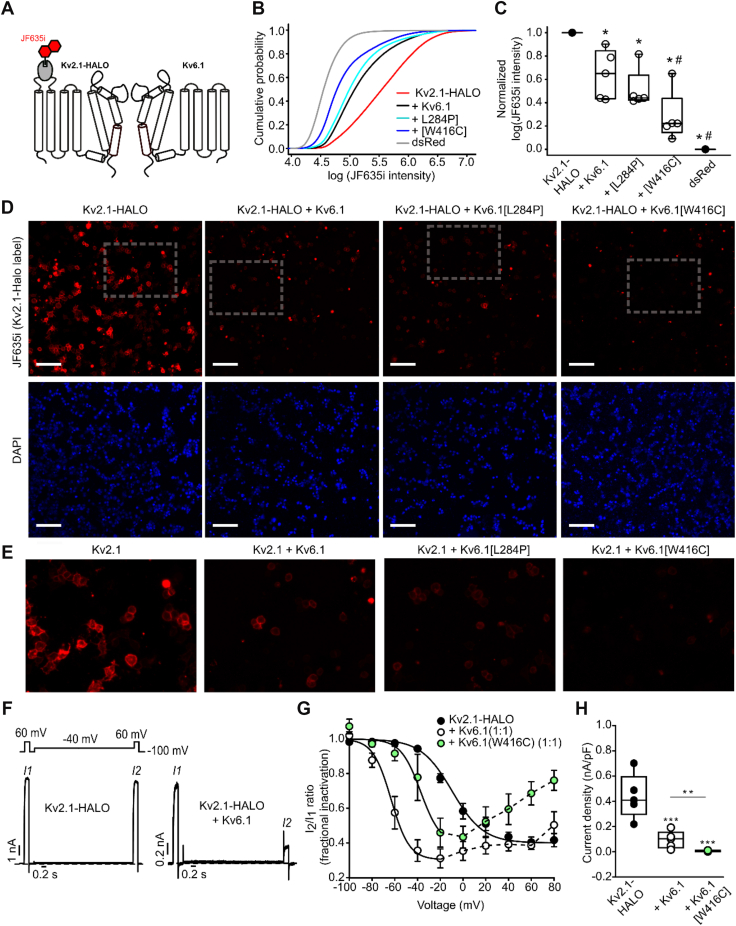
**HALO-label detection of cell surface Kv2.1 expression.***A*, schematic of a HALO-Kv2.1: Kv6.1 assembly. Membrane-impermeant Halo dye JF635i (shown in *red*) reacts with the HaloTag in the extracellular S1-S2 linker of Kv2.1 at the cell surface. *B*, cumulative probability of JF635i integrated intensity from individual cells in each transfection condition (five independent experiments). *C*, log of integrated intensity of the HaloJF635i normalized to the Kv2.1 alone group (max value, 1) and the dsRed alone group (min value, 0), for indicated combinations of Kv2.1 and Kv6.1 variants. A repeated measures ANOVA (on the raw median log JF635i integrated intensity in each experiment) followed by a Tukey’s *post hoc* test was used to compare experimental groups where appropriate (∗ indicates *p* < 0.05 relative to HALO-Kv2.1 alone and # indicates *p* < 0.05 relative to the HALO-Kv2.1:Kv6.1 WT condition). *D*, HEK293 cells were transfected with combinations of HALO-Kv2.1 and Kv6.1 (WT or variants) as indicated, labeled with membrane-impermeant Halo-JF635i, and cell-by-cell fluorescence intensity was quantified using high content microscopy. The scale bar (in *white*) represents 100 μm. *E*, insets from *panel D* Halo-JF635i label for Kv2.1 alone and with Kv6.1 variants as indicated. *F*, exemplar sweeps of HALO-Kv2.1 and Kv6.1 with a −40 mV conditioning pulse to assess inactivation (as done in Fig. 2*E*). *G*, voltage dependence of inactivation of HALO-Kv2.1 with Kv6.1 variants (as measured in Fig. 2*F*, n = 3–6 per group). *H*, current density at +30 mV for indicated subunit combinations (1:1 cDNA ratio) after transient transfection in LM cells. One-way ANOVA followed by Tukey *post hoc* test was used to compare experimental groups, with ∗∗∗ and ∗∗ indicating *p* < 0.001 and *p* < 0.01, respectively, n = 6 per group. The control groups in all experiments were cotransfected with soluble GFP to ensure a constant amount of plasmid DNA in the transfection mixture.

We developed a method to assess expression of Kv2.1 channels using high content microscopy, based on a cell surface labeling approach using HALO tags. We inserted a HALO tag into the extracellular S1-S2 linker of Kv2.1, at residue 221 (Fig. 6*A*). The function of this epitope-tagged channel was validated using electrophysiology (Fig. 6, *F*–*H*). The Kv2.1-HALO construct exhibits inactivation properties closely resembling WT Kv2.1 (Fig. 6*G*). Kv2.1-HALO channels also exhibit prominent modulation by Kv6.1 in terms of reduced currents and shifted inactivation properties (Fig. 6, *F*–*H*). Similar to WT Kv2.1, coexpression of Kv6.1[W416C] also leads to blunted effects on inactivation when compared to WT Kv6.1 (Fig. 6*G*). We also confirmed that Kv2.1-HALO current density is reduced by Kv6.1 and even more dramatically by Kv6.1[W416C] (Fig. 6*H*). These experiments are consistent with our findings for WT Kv2.1, indicating that Kv2.1-HALO retains responsiveness to Kv6.1 and candidate disease-linked variants.

Using membrane-impermeant HALO dyes, we assessed cell surface expression levels of Kv2.1 alone and when coexpressed with Kv6.1 or various mutants (as in previous experiments, cotransfection with soluble GFP was used to ensure a constant amount of plasmid DNA in the transfection mixture). Figure 6*D* (expanded view in Fig. 6*E*) illustrates sample images of HaloJF635i-labeled channels (red) and 4′,6-diamidino-2-phenylindole (DAPI) staining (blue, used to identify cells during quantification). Findings from these images are quantified with a cumulative probability distribution of the integrated intensity of JF635i (membrane-impermeant fluorescent HALO tag) in the indicated combinations of Kv2.1, Kv6.1, and Kv6.1 variants (Fig. 6, *B* and *C*). The cumulative probability plot (Fig. 6*B*) indicates the likelihood of a randomly selected cell to exhibit a given integrated intensity (or lower). Curves that fall further left on the *x*-axis indicate a higher likelihood of cells with a low integrated JF635i intensity, whereas curves further to the right indicate that cells are more likely to exhibit a higher integrated intensity. As this method measures fluorescence from several 1,000 cells per well, this type of plot is helpful to illustrate the distribution of intensity of the Kv2.1-HALO signal among transfected cells, and the effects of coexpression with Kv6.1 variants. Consistent with Western blot findings, these experiments illustrate robust Kv2.1 expression at the cell surface when expressed alone, but attenuated surface expression when coexpressed with Kv6.1 or Kv6.1[L284P], and even further suppression when coexpressed with Kv6.1[W416C] (Fig. 6, *B* and *C*).

### Stoichiometric interplay between Kv2.1 and Kv6.1

**Figure 7:**
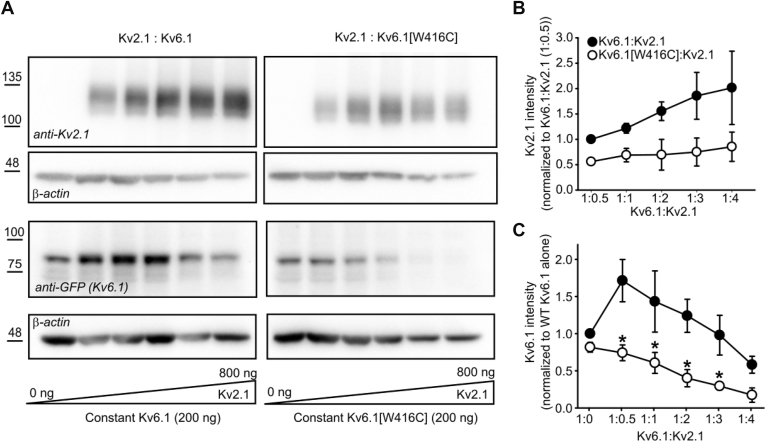
**Stoichiometric interplay affects expression of Kv6.1 and Kv2.1.***A*, HEK293 cells were transfected with a constant (200 ng) amount of Kv6.1 (*left*) or Kv6.1[W416C] (*right*), and increasing amounts of Kv2.1 (0, 100 ng, 200 ng, 400 ng, 600 ng, 800 ng) for 72 h, and probed for Kv2.1, Kv6.1, or β-actin, as indicated (n = 3). The groups were cotransfected with varying amounts of soluble GFP to ensure constant plasmid DNA in the transfection mixture. *B*, Kv2.1 bands were normalized to the Kv2.1 signal from the 1:0.5 (WT Kv6.1:Kv2.1) ratio condition. *C*, Kv6.1 bands were normalized to the Kv6.1 signal from the 1:0 (Kv6.1:Kv2.1) ratio condition. A paired *t* test was used to compare Kv6.1 *versus* Kv6.1[W416C] expression at each ratio (∗ indicates *p* < 0.05, *panel C*). Data in (*B*, *C*) are expressed as mean ± SEM from three independent experiments.

As there is uncertainty about the stoichiometry of assembly of KvS subunits with Kv2 subunits, we investigated varying ratios of Kv2.1 to Kv6.1 (or Kv6.1[W416C]). We first tested transfection of a constant amount of Kv6.1 (WT or [W416C]) together with increasing amounts of Kv2.1 (Kv6.1:Kv2.1 ratios of 1:0.5, 1:1, 1:2, 1:3, and 1:4), balanced by GFP to ensure a constant amount of DNA in the transfection. The purpose of this experiment was to determine whether the stoichiometric balance of Kv6.1 and Kv2.1 could alter the suppressive effects of Kv6.1. All ratios of Kv6.1[W416C] strongly diminished Kv2.1 expression relative to WT Kv6.1, even at the highest amounts of Kv2.1 tested (Fig. 7*A*, upper panels; Fig. 7*B*). This indicates that in the range of conditions sampled, the suppressive effects of Kv6.1[W416C] were not saturated or surmounted by increasing levels of Kv2.1 (up to a 1:4 cDNA ratio). We also assessed Kv6.1 expression in this experiment (Fig. 7*A*, lower panels; Fig. 7*C*). WT Kv6.1 exhibited stabilization by intermediate levels of Kv2.1, but declined at higher ratios of Kv2.1, suggesting there is an optimal ratio that can support expression of both Kv2.1 and Kv6.1. In contrast, increasing Kv2.1 led to a progressive decline of Kv6.1[W416C] at all ratios tested. This further supports our finding reported in Figure 4, showing that reduced expression of Kv6.1[W416C] is driven by coexpression with Kv2.1 (whereas the W416C mutation does not have a prominent impact when expressed in the absence of Kv2.1). The most clear explanation of these findings is that by increasing Kv2.1 levels, there is an increased likelihood of heteromeric assembly with Kv6.1[W416C], leading to further reduction of Kv6.1[W416C] expression (Fig. 7*C*).

**Figure 8:**
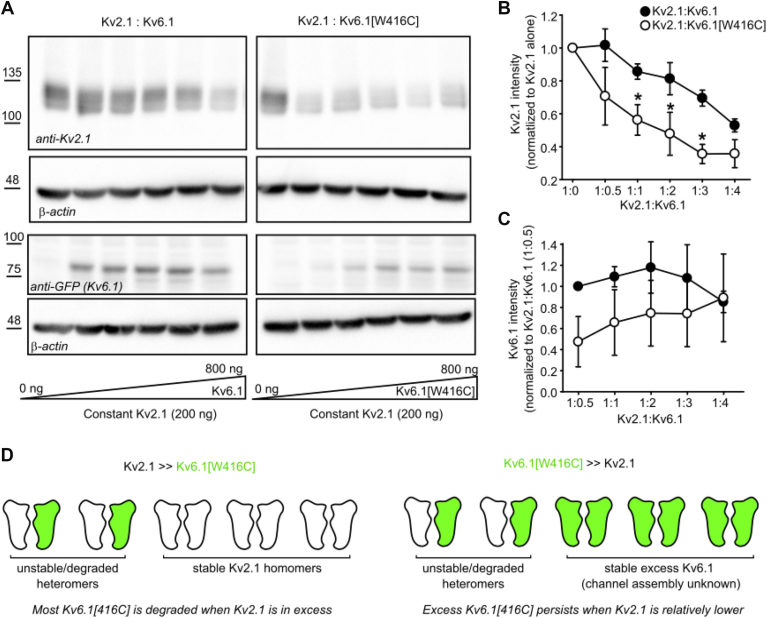
**Kv2.1 coassembly underlies suppression of Kv6.1[W416C].***A*, HEK293 cells were transfected with a constant (200 ng) amount of Kv2.1 and increasing amounts of WT Kv6.1 (*left*) or Kv6.1[W416C] (*right*), (Kv6.1 cDNA amounts: 0, 100 ng, 200 ng, 400 ng, 600 ng, 800 ng) for 72 h, and probed for Kv2.1, Kv6.1, or β-actin, as indicated (n = 3). The groups were cotransfected with soluble GFP wherever applicable to ensure a constant amount of plasmid DNA in the transfection mixture. Protein ladder molecular weights are in kDa. *B*, Kv2.1 signal density was normalized to Kv2.1 signal from the 1:0 (Kv2.1:Kv6.1) ratio condition. A paired *t* test was used to compare Kv2.1 coexpressed with either Kv6.1 or Kv6.1[W416C] expression at each ratio (∗ indicates *p* < 0.05, *panel B*). Data are expressed as mean ± SEM from three independent experiments. *C*, Kv6.1 signal density was normalized to WT Kv6.1 signal from the 1:0.5 (Kv2.1:Kv6.1) ratio condition. Data are expressed as mean ± SEM from three independent experiments. *D*, schematic diagram indicating stability of Kv2.1 and Kv6.1[W416C] heteromers or homomers of each channel under either excess Kv2.1 or excess Kv6.1[W416C] compared to the other one.

We carried out a similar experiment to investigate Kv6.1:Kv2.1 ratios, but this time transfected cells with a constant amount of Kv2.1, and progressively increased the amount of cotransfected Kv6.1 (WT or [W416C], balanced with GFP to maintain a constant amount of cDNA in the transfection, Figure 8). In this case, we found that increasing cotransfected amounts of Kv6.1[W416C] caused a strong decline in Kv2.1 expression (Fig. 8*A*, top right panel; 8B), *versus* WT Kv6.1. We also verified Kv6.1 expression in each case (Fig. 8*A*, lower panels; 8C). WT Kv6.1 expression remained relatively consistent at all tested amounts. Kv6.1[W416C], as expected, exhibited lower expression than WT Kv6.1 when it was in a low stoichiometric ratio relative to Kv2.1. However, transfection with higher amounts of Kv6.1[W416C] could overcome this effect and lead to higher Kv6.1[W416C] when it was in stoichiometric excess relative to Kv2.1. In general, this is also consistent with the demonstration that heteromerization with Kv2.1 is the primary driver of weakened Kv6.1[W416C] expression (Fig. 4). Thus, as the amount of transfected Kv6.1[W416C] is increased, it may saturate available Kv2.1 subunits, leading to a higher amount of Kv6.1[W416C]. These overall findings (Figs. 7 and 8) are summarized in Figure 8*D*: when Kv2.1 expression is in excess relative to Kv6.1[W416C], there is a strong likelihood that W416C subunits will assemble with Kv2.1 and be suppressed/degraded. However, when there is an excess of Kv6.1[W416C], many subunits will not assemble with Kv2.1, and thus not be degraded. At present it is unclear how/whether these excess Kv6.1[W416C] subunits are assembled into tetramers, but their signal clearly persists on Western blots (*e.g.*, Fig. 8*A* lower right panel, at high Kv6.1[W416C] transfection amount). Overall, these findings further highlight that the apparent effects of the disease-linked Kv6.1 mutants are accentuated when coexpressed with Kv2.1 and may not be apparent when the Kv6.1 mutant subunit is expressed alone.

### Phosphorylation in Kv2.1/Kv6.1 protein dynamics

**Figure 9:**
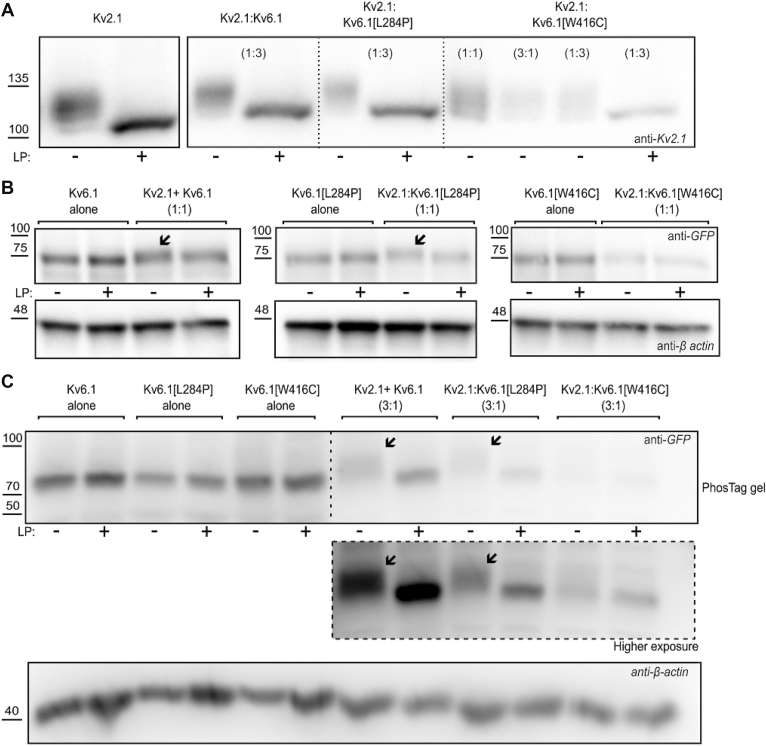
**Coassembly with Kv2.1 underlies Kv6.1 phosphorylation.***A*, Kv2.1 was coexpressed with Kv6.1 variants in HEK293 cells for 72 h. Lysates were treated with Lambda phosphatase (LP) as indicated, separated by SDS-PAGE and probed with an anti-Kv2.1 antibody (n = 3). *B*, Kv2.1 and Kv6.1 variants were cotransfected in HEK293 cells in 1:1 ratio. Lysates were treated with LP as indicated, separated by SDS-PAGE, and probed with an anti-GFP antibody (n = 3). *Arrows* denote shifted mobility of Kv6.1 and Kv6.1[L284P] when coexpressed with Kv2.1. Mobility shifts are not seen for Kv6.1 (or variants) when expressed alone. *C*, Kv2.1 and Kv6.1 variants were cotransfected in HEK293 cells, in 3:1 (Kv2.1:Kv6.1) ratio. Lysates were treated with LP as indicated, separated by a 100 μM phos-tag SDS-PAGE gel, and probed with an anti-GFP antibody (n = 2 of 3:1 ratio and n = 4 for Kv2.1: Kv6.1 in 1:1 ratio, not shown here). Phosphorylated Kv6.1 and Kv6.1[L284P] bands are highlighted with *arrows*. A higher exposure is shown in the *dotted box* for clearer depiction of phosphorylated bands. Anti–β-actin was used as a loading control in *panels A and B*. The control groups (individual subunits expressed alone) were cotransfected with soluble GFP to ensure a constant amount of plasmid DNA in all experiments.

Several studies have shown a role for activity dependent Kv2.1 phosphorylation in fine control of neuronal excitability and plasticity, and Kv2.1 phosphorylation can be readily detected with Western blot approaches (42, 43, 44). However, posttranslational modifications of Kv6.1 subunits have not been described. Based on the altered mobility of Kv6.1 when coexpressed with Kv2.1 (see Fig. 4), we investigated the contributions of phosphorylation. We first validated our approach by demonstrating Kv2.1 phosphorylation in various homomeric and heteromeric channel combinations, using Lambda phosphatase treatments (Fig. 9*A*). Lambda phosphatase cleaves phosphorylation groups from Thr, Ser, and Tyr sidechains, causing a band shift of Kv2.1 to a lower molecular weight, slightly above 75 kDa (Fig. 9*A*). These blots illustrate that Kv2.1 (alone or coexpressed with Kv6.1) is predominantly phosphorylated in the heterologous HEK293 system used for these experiments, and this was not markedly affected by coexpression with Kv6.1 or Kv6.1[L284P]. Coexpression with Kv6.1[W416C] weakens overall expression of Kv2.1, along with a greater fraction of channels at the “unphosphorylated” molecular weight (Fig. 9*A*, most apparent in the 1:1 transfection condition).

Since the posttranslational modifications of Kv6.1 have not been previously described, we investigated the phosphorylation state of Kv6.1, and how it is affected by Kv2.1, based on shifts in band mobility. In experiments described earlier, there was a consistent shift in migration of Kv6.1 and Kv6.1[L284P] when coexpressed with Kv2.1 (Fig. 4*A*), although this was not as pronounced as for Kv2.1 (*e.g.*, Fig. 9*A*). We also found that these Kv2.1-dependent mobility shifts of EGFP-Kv6.1 are Lambda phosphatase sensitive (Fig. 9*B*), although the effects on mobility are not dramatic. Therefore, we used Phos-Tag acrylamide gels to accentuate the altered mobility of phosphorylated proteins, and this approach was more effective at demonstrating clear Lambda phosphatase-sensitive mobility shifts of EGFP-Kv6.1 when coexpressed with Kv2.1 (Fig. 9*C*, higher exposure included as an inset, due to the lower expression of Kv6.1 when coexpressed with Kv2.1). In this case, based on the stoichiometry dependence observed in Figures 7 and 8, we used an excess of Kv2.1 (3:1 Kv2.1:Kv6.1) to promote assembly of Kv2.1:Kv6.1 heteromers and thus increase the likelihood of Kv2.1-dependent modifications of Kv6.1. The mobility shift was observed for both Kv6.1 and Kv6.1[L284P], indicating the involvement of phosphorylation (Fig. 9, *B* and *C*). This highlights a previously unreported phosphorylated form of Kv6.1 (and Kv6.1[L284P]), which is favored only when Kv2.1 is present. Interestingly, this higher migrating phosphorylated isoform appears to be absent in Kv6.1[W416C] whether Kv2.1 was coexpressed or not (Figs. 4*A*, and 9, *B* and *C*). This, together with weakened phosphorylation of Kv2.1 (Fig. 9*A*) indicates that Kv6.1[W416C] likely engages with Kv2.1 early in biogenesis, prior to the phosphorylation events that are coupled to maturation of Kv2.1.

### Kv6.1 modulation of Kv2.2

**Figure 10:**
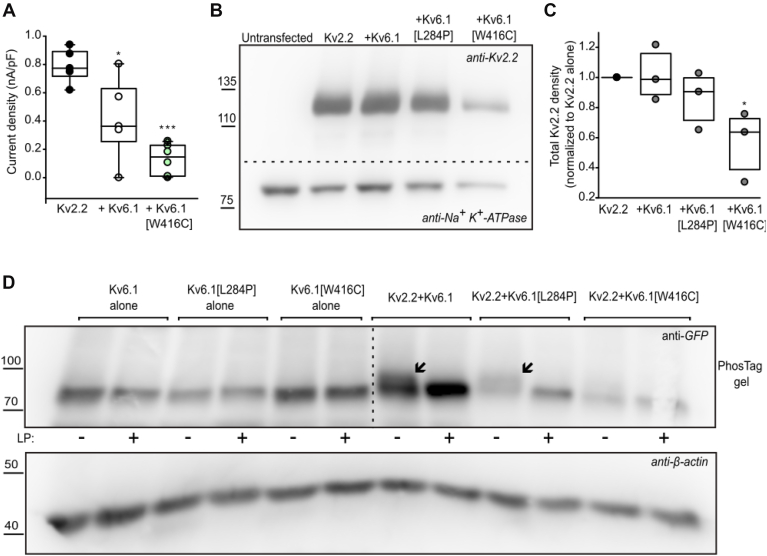
**Common features of Kv2.2 and Kv2.1 modulation by Kv6.1.***A*, current density at +30 mV was measured from indicated subunit combinations (1:1 cDNA ratio of Kv2.2 and Kv6.1 WT or Kv6.1[W416C]) after transient transfection in LM cells. Control groups were cotransfected with soluble GFP to ensure a constant amount of plasmid DNA in the transfection mixture in all experiments. One-way ANOVA followed by Tukey *post hoc* test was used to compare groups (∗ indicates *p* < 0.05; ∗∗ indicates *p* < 0.001), n = 6. *B*, mOrange-Kv2.2 was coexpressed with Kv6.1, Kv6.1[L284P], and Kv6.1[W416C] in 1:1 ratio for 72 h in HEK293 cells. Whole-cell lysates were separated on SDS-PAGE and probed with anti-Kv2.2 and Na^+^/K^+^-ATPase (loading control) antibodies. *C*, Kv2.2 signal in each group from *panel B* is plotted as normalized to the Kv2.2 control (channel expressed alone) (n = 3). A repeated measures ANOVA test (on raw Kv2.2 density normalized to β-actin) followed by a *post hoc* paired *t* test was used to compare experimental groups (∗ indicates *p* < 0.05 relative to Kv2.2 alone). *D*, Kv6.1 or variants were expressed alone or with Kv2.2 (1:1 ratio), for 72 h in HEK293 cells. Whole-cell lysates were separated by phos-tag SDS-PAGE and probed using an anti-GFP (β-actin loading control (n = 3)). *Arrows* indicate the slower migration of phosphorylated forms of Kv6.1 and Kv6.1[L284P] when coexpressed with Kv2.2 (representative blot from three experiments).

Based on recent findings describing Kv2.2 variants in a syndrome involving craniofacial and limb dysmorphisms, along with some cases of neurodevelopmental delay, we also carried out preliminary exploration of Kv6.1 modulation of Kv2.2 (29). Kv2.2 current density was modestly attenuated by Kv6.1 coexpression, whereas Kv6.1[W416C] retained the strong current suppression of Kv2.2 (Fig. 10*A*). Kv6.1[W416C] also prominently suppressed Kv2.2 expression (Fig. 10, *B* and *C*), although Kv6.1 and Kv6.1[L284P], did not significantly alter Kv2.2 expression when cotransfected at 1:1 ratio (Fig. 10, *B* and *C*). Kv2.2-dependent posttranslational modifications of Kv6.1 and Kv6.1[L284P] were also apparent (Phos-Tag gel in Fig. 10*D*), but did not occur with Kv6.1[W416C]. This feature is consistent with phosphorylation observed for Kv6.1 and Kv6.1[L284P] in complex with Kv2.1 (Fig. 9, *B* and *C*). Overall, these preliminary observations hint at common outcomes and mechanisms of Kv2.1 and Kv2.2 association with Kv6.1 WT, although the relative importance of assembly with Kv2.1 *versus* Kv2.2 remains uncertain.

## Discussion

Kv2.1 plays a multifaceted role in normal human physiology and disease states. In addition to shaping moment-to-moment electrical signaling, Kv2.1 is crucial for neuronal development, influences postinjury neurons in neurodegenerative disorders, and other potential roles (27, 45). Ion channels have a crucial role in regulating emergent neural circuits during early brain development and Kv2.1 mutations have been shown to cause morphological and synaptic abnormalities arising during neocortex development (46). *KCNB*/Kv2 family channels are considered unique in their ability to heterotetramerize with an array of silent Kv subunits, which contributes to further functional diversity of these channels and involvement in diverse cellular functions. For example, Kv9.1 facilitates Kv2.1 function, and its reduction following traumatic nerve injury leads to mechanical hypersensitivity (47, 48). There are also examples of involvement of silent Kv channels in developmental disorders. For instance, a Kv6.4 mutation has been linked to impaired development of the brain ventricular system (49). Kv8.2 heterozygous variants have been implicated in development of retinal disorder cone dystrophy with supernormal rod responses through its interaction with Kv2.1 (50).

In this study, we investigated the interplay between Kv2.1 and two uncharacterized variants of Kv6.1, identified in organisms with musculoskeletal anatomical abnormalities, sometimes accompanied by other symptoms. We reported a pediatric patient carrying the Kv6.1[L284P] mutation who exhibited limb abnormalities, along with other features, but generally normal neurodevelopment. A previously published report of the [W416C] mutation in a calf described craniofacial dysmorphism, accompanied by neuromuscular degeneration leading to muscle stiffness, and a form of Hyperkalemic Periodic Paralysis (34), but the authors did not comment on limb development. Although the link between ion channel function and anatomical development is not yet clear, other channels have also been reported to influence these features, with hand anomalies often observed. For example, *KCNJ2*/Kir2.1 mutations have been linked to musculoskeletal abnormalities in Andersen–Tawil syndrome (51, 52). Additionally, *KCNH1* variants have also been linked to Temple–Baraitser syndrome and Zimmermann–Laband syndrome, both of which are characterized by features like nail dysplasia, facial dysmorphism, and intellectual disability, while Zimmermann–Laband syndrome are further associated with hypoplasia of distal phalanges (53, 54, 55, 56). Perhaps most interestingly in the context of the effects of Kv6.1 mutations is the recent report that mutations in Kv2.2 have been identified in a cohort of patients exhibiting variable craniofacial and hand dysmorphisms along with neurodevelopmental delay in some cases, thus there may be some overlapping phenotypic features of Kv2.2 and Kv6.1 variants (29). These Kv2.2 variants exhibit diminished function when expressed alone, either by lowering current or by increased extent of inactivation (29), and our preliminary findings indicate that there are commonalities in Kv2.2 and Kv2.1 modulation by Kv6.1. While future studies are certainly required to establish causality, disease mechanism, and the interplay between Kv6.1 and Kv2.1 (or Kv2.2), there are interesting overlapping features and regulatory properties of these channel subtypes. One challenge for developmental studies of ion channels in the context of limb/bone development is that there are varying reports of *KCNG1*/Kv6.1 transcript abundance in bone or osteogenic precursor cells ((57, 58), Genevestigator database). However, other K^+^ channels (*e.g.*, *KCNJ2*, *KCNH1*) with well-established links to dysmorphic traits frequently exhibit low transcript abundance. Thus we have not assessed cell types or developmental stages where these channel variants could exert their influence. Future investigations could implement approaches such as RNAscope to investigate, colocalization of Kv2.1/Kv2.2/Kv6.1 mRNAs can be studied in bone at different developmental stages.

More cases are certainly needed to link *KCNG1* to limb anomalies or other skeletal deformities, and we emphasize that direct causal relationships in the cases described here are not clear, especially in the case of the relatively weak functional outcomes described for the L284P mutation. Limb-related genetic diseases are often variably penetrant and thus a clear role of *KCNG1* might be difficult to sort out as other modifiers may be present. It is impractical to search for modifiers in this case (due to lack of sample size), but we note that the other notable variant in the human patient was the deletion in intron 1 of *ANKRD11* (also present in the unaffected father). This is a variant of unknown significance, which we suspect to not affect protein coding but could have unknown regulatory impacts or converge with Kv6.1 by some unidentified mechanism. For example, one possibility is that absence of the first intron could interfere with transcription initiation or lead to partial loss of function of *ANKRD11*, in turn affecting Kv6.1 regulation. Alternatively, subtle changes in Kv2.1 inactivation mediated by Kv6.1[L284P] could influence electrical signaling during skeletal development and exacerbate otherwise mild effects of the *ANKRD11* intronic deletion.

Both the gating and expression effects of Kv6.1 mutants could influence phenotypic outcomes. We observed graded effects of Kv6.1[L284P] and Kv6.1[W416C] on the inactivation properties of Kv2.1, as each mutant weakens Kv6.1-mediated inactivation properties (Figs. 2*G*, and 6). In terms of expression, the L284P mutation did not have consistent or convincing differences relative to WT Kv6.1, whereas the W416C mutation caused powerful suppression of channel expression when coexpressed with Kv2.1. As mentioned, an interesting outcome of these findings is that the mutations themselves do not appear to be significantly destabilizing, but their effects become apparent when coexpressed with Kv2.1. This likely highlights a regulatory pathway that requires coassembly of Kv6.1 and Kv2.1, and this raises further questions related to proteostasis of heteromeric Kv2/KvS channels. For example, based on the recent reports of Kv2.2-linked pathologies, it will be worthwhile to carry out a more detailed characterization of functional interactions between Kv2.2 and Kv6.1. Our preliminary findings suggest a similar impact of Kv2.2 and Kv6.1/variant association, among which the mutual effects of Kv6.1[W416C] and Kv2.2 assembly mimics that of Kv2.1 with the same variant. In addition, questions persist about the cellular pathways that influence expression of Kv2.x:Kv6.1 heteromeric channels, and whether their expression levels are controlled at the level of maturation, or by a degradation pathway. Lastly, close attention has been devoted to the preferred stoichiometry of Kv2:Kv6 heteromers (37, 38), and so identification of the suppressive effect of Kv6.1[W416C] may be a useful tool for determining the subunit composition required to cause profound suppression of heteromeric channel expression.

We also observed mutant-dependent and assembly-dependent alterations of posttranslational modifications, of both Kv2.1 and Kv6.1 (Fig. 9). These findings indicate that while Kv2.1 alone is readily phosphorylated, Kv6.1 phosphorylation occurs when coassembled with Kv2.1, suggesting that Kv2.1 may underlie recruitment of the kinases involved. Kv6.1 and Kv6.1[L284P] phosphorylation states observed in presence of Kv2.2 (Fig. 10*D*) potentially suggest a similar mode of kinase recruitment by Kv2.2 as well. Posttranslational modification of Kv2.1 by phosphorylation has been extensively studied. Kv2.1 is directly phosphorylated by CDK5, which maintains Kv2.1 at constitutively highly phosphorylated state in neurons (42). Other studies have shown that AMPK-dependent phosphorylation of Kv2.1 has implications on neuronal metabolic stress (43) and dual phosphorylation at N-terminal Y124 and C-terminal S800 by Src and p38, respectively, is required in proapoptotic Kv2.1 membrane insertion in injured neurons (59). Kv2.1 has been found to be dephosphorylated by protein phosphatase-1, which is initiated by N-methyl-D-aspartate receptors in response to synaptic activity (60). In terms of phosphorylation of Kv6.1, far less is known, although early studies described potential phosphorylation sites (by PKA, CaMKII, or CKII) that might undergo modification after coassembly with Kv2.1 (61).

In summary, we report the functional characterization of two candidate disease-linked variants of Kv6.1/*KCNG1*. These variants exhibit distinct effects on expression, posttranslational modifications, and gating of Kv2.1, with the effects of W416C being far more prominent than L284P overall. Further mechanistic investigation and larger numbers of human cases will be required to establish a clear causal relationship between Kv6.1 mutations and anatomical abnormalities. However, our findings contribute to the understanding of mechanisms controlling Kv2/KvS assembly, posttranslational modifications, proteostasis, and gating.

## Experimental procedures

### Cell culture

Cell culture was maintained at 37 °C in a 5% CO_2_ incubator in Dulbecco's modified Eagle's medium supplemented with 10% fetal bovine serum and 1% Penicillin/Streptomycin. Cells were transfected with cDNA using jetPRIME transfection reagent (Polyplus). HEK293 cells American type culture collection (ATCC) were used for Western blot and high content microscopy experiments. Mouse L(tk-) fibroblast cell line (ATCC CCL-1.3), referred to throughout as LM cells, were used for whole-cell patch-clamp studies, because they lack endogenous voltage-gated K^+^ currents.

### Plasmid constructs and mutagenesis

All cDNAs were expressed in pcDNA3.1(−) vector (Invitrogen). Kv2.1 was obtained from Addgene (plasmid #131707) and expressed in pIRES2-DsRed-MST (Clontech), while Kv6.1 and Kv2.2 were provided by DNASU (clone #HsCD00877145 and clone #HsCD00080393, respectively). HALO-Kv2.1 was created by PCR amplification of the modified haloalkane dehydrogenase enzyme (HALO), with the following primers: HALO-Kv2.1-5′: gggggtccggaatggcagaaatcggtactg, HALO-Kv2.1-3′: gggggtccggagccggaaatctcgagcgtc. This fragment was inserted into a unique BspEI site previously introduced at position Gly221 in the Kv2.1 construct, using restriction digest and ligation followed by screening for correct orientation of the insert. Kv2.2 was cloned into pLSSmOrange-C1 (Addgene) plasmid using EcoRI and KpnI restriction sites following PCR amplification with the following primers: Kv2.2-EcoRI-5′: ggggaattctatggcagaaaaggctcccccg, Kv2.2-KpnI-stop-3′: gggggtacctcacatgctggtttcacgtgt, and this clone has been used for Kv2.2-related experiments. Site-directed mutagenesis in KCNG1/Kv6.1 was carried out using an overlap extension PCR. Kv6.1[L284P] and Kv6.1[W416C] mutants were constructed using NheI and HindIII restriction sites using the following primers and inserted in pcDNA3.1(−). Mutagenic primers used were as follows: Kv6.1[L284P] 5′: ctg gag ttc ccc ctg cgg ctc, Kv6.1[L284P] 3′: gag ccg cag ggg gaa ctc cag; Kv6.1[W416C] 5′: gcc tgc tac tgt tgg gct gtc, Kv6.1[W416C] 3′: gac agc cca aca gta gca ggc. EGFP was fused with the Kv6.1 constructs at the N terminus using standard PCR with NheI and XhoI restriction enzyme sites, compatible restriction digestion and ligation. Constructs were verified using diagnostic restriction digestion, followed by Sanger sequencing (University of Alberta Applied Genomics Core). For all Western blot and electrophysiology experiments, the Kv2.1 to EGFP-6.1 constructs as well as mOrange-Kv2.2 to EGFP-6.1 constructs were expressed in 1:1 ratio unless otherwise noted. Total DNA was kept constant in all conditions within each experiment using GFP plasmid.

### Lambda phosphatase treatment

Lambda phosphatase treatment was used to remove phosphate groups from proteins in cell lysates. Fifty micrograms of protein lysate was incubated with 5 μl of NEB buffer for protein metallophosphatases, 5 μl of 10 mM MnCl_2_ and 1 μl of Lambda Protein Phosphatase. The mixture was incubated at 30 °C for 30 min, followed by Western blot.

### Western blot

Cells were passaged into 12-well plates and transfected with desired combinations of plasmids. Cell lysates were collected in NP-40 lysis buffer (1% NP-40, 150 mM NaCl, 50 mM Tris–HCl) and 1% Protease Inhibitor cocktail (Sigma-Aldrich, P8340), 72 h posttransfection. Samples were separated using 8% SDS-PAGE gels and then transferred onto a nitrocellulose membrane using a standard Western blot apparatus (Bio-Rad). Kv2.1 was detected using a mouse monoclonal Kv2.1 antibody (1:850 dilution; clone (K89/34) 75-014, NeuroMab) and Horseradish peroxidase (HRP)-conjugated goat anti-mouse antibody (1:30,000 dilution; HS023, Applied Biological Materials). Kv2.2 was detected with a mouse monoclonal Kv2.2 antibody (1:1000 dilution; clone (N372B/1) 75-369, NeuroMab) and HRP-conjugated goat anti-mouse antibody (1:30,000 dilution; HS023, Applied Biological Materials). Kv6.1 was detected using goat polyclonal anti-GFP (1:1000; G0950, Applied Biological Materials) and HRP-conjugated donkey anti-goat antibody (1:25,000; SH011, Applied Biological Materials). β-Actin or Na^+^/K^+^-ATPase were used as loading control and detected using the β-actin mAb (1:10,000 dilution; GT5512, GeneTex) and HRP-conjugated goat anti-mouse antibody (1:30,000 dilution; HS023, Applied Biological Materials) or anti-Na^+^/K^+^-ATPase antibody (1:1000 dilution; 3010S, Cell Signaling Technology) and HRP-conjugated goat anti-rabbit antibody (1:20,000; ab6721, Abcam), respectively. Chemiluminescence was detected using a SuperSignal West Femto Max Sensitivity Substrate (Thermo Fisher Scientific) and a ChemiDoc imaging system (Bio-Rad).

### Phos-tag SDS-PAGE

Phosbind acrylamide (final concentration 100 μM; F4002, APExBIO) and MnCl_2_ (final concentration 400 μM) were added to a 8% resolving gel for a phos-tag SDS-PAGE gel assay. An EDTA-free protein ladder (F4005, APExBIO) was run alongside the samples. Before transfer onto a PVDF membrane, manganese ions were removed from the gel by washing the gel for 20 min each (three times) in a transfer buffer containing 10 mM EDTA, followed by two washes in an EDTA-free transfer buffer for 10 min each.

### Coimmunoprecipitation

HEK293 cells were lysed 72 h posttransfection and collected in a NP-40 lysis buffer supplemented with 1% protease Inhibitor cocktail (P8340, Sigma-Aldrich). Two hundred fifty microliters of NP-40 lysis buffer containing 500 μg of protein lysate was incubated with 1 μl of 100 μg/ml concentration of either GAPDH (ab9485, Abcam) or Kv2.1 (75-014, NeuroMab) or GFP (G0950, Applied Biological Materials) antibodies overnight at 4 °C. Protein A/G plus agarose beads (40 μl) (SC-2003; Santa Cruz) were added to the lysate containing the antibodies for 2 h on a rotator at 4 °C. Beads were centrifuged at 9600*g* for 1 min and then washed five times with cold NP-40 lysis buffer. The beads were resuspended in a 6X loading buffer and were boiled for 5 min, followed by centrifugation at 9600*g* for 5 min. Immunoprecipitates were separated using SDS-PAGE gels and subjected to Western blot analysis. Kv2.1 and EGFP-Kv6.1/variants were detected using mouse monoclonal Kv2.1 antibody (75-014, NeuroMab), followed by HRP-conjugated goat anti-mouse antibody (SH023, Applied Biological Materials), and goat polyclonal anti-GFP (G0950, Applied Biological Materials) followed by HRP-conjugated donkey anti-goat antibody (SH011, Applied Biological Materials) respectively.

### Bioluminescence resonance energy transfer

A Nanoluc-Kv2.1 fusion protein (NanoLuc luciferase; BRET donor) was generated by PCR amplification with the following primers: Kv2.1-NotI-5′: gggggcggccgctaatgccggcgggcatgacg, Kv2.1-KpnI-stop-3′: ggggggtacctcagatgctctgatctcg, followed by ligation with compatible restriction sites NotI and KpnI using pcDNA3.1-ccdB-Nanoluc as the Nanoluc template (gift from Mikko Taipale, Addgene plasmid #87067). mEGFP tagged at the N terminus of Kv6.1 or the Kv6.1 variants was used as BRET acceptor. LM cells were transiently transfected with BRET donor and acceptor cDNAs for 48 h, followed by replating onto white polystyrene 96-well plates (Thermo Fisher Scientific). Post 24 h, cells were washed with 1X PBS, and incubated with Nano-Glo live-cell assay reagent (Promega). Emission spectra were measured for each pair between 400 and 700 nm in 5 nm increments, for 2 s at each interval, using a MiniMax plate reader (Molecular Devices). Emission spectra were normalized to the peak nanoluc emission in each well.

### Electrophysiology

Patch pipettes were made from soda lime capillary glass (Fisher), using a Sutter P-97 puller (from Sutter instrument). With standard internal solution, the tip resistance was between 1 to 3 MΩ. Recordings were filtered at 5 KHz and sampled at 10 KHz, with manual capacitance compensation and series resistance compensation between 70 to 80%. The data were stored using Clampex software (Molecular Devices; https://www.moleculardevices.com/products/axon-patch-clamp-system/acquisition-and-analysis-software/pclamp-software-suite). The external (bath) solution composition was 135 mM NaCl, 5 mM KCl, 1 mM CaCl_2_, 1 mM MgCl_2_, 10 mM Hepes, and the pH was adjusted to 7.3 using NaOH. The internal pipette solution composition was 135 mM KCl, 5 mM K-EGTA, 10 mM Hepes, and the pH was adjusted to 7.2 using KOH. For electrophysiological recordings, cells were initially plated in 12-well plates and replated onto coverslips 6 h after transfection. Recordings were done 24 h posttransfection.

### Labeling of cells for high content microscopy

HEK293 cells were passaged into 6-well plates and transfected with 1 μg of Halo-Kv2.1 and either 1 μg of EGFP, EGFP-Kv6.1[WT], EGFP-Kv6.1[L284P], or EGFP-Kv6.1[W416C] for 48 h (pIRES2-DsRed-MST empty vector transfected cells were used as a negative control). Transfected cells were then seeded onto a 96-well μ-clear black-walled plate (Greiner, 655090) for 24 h followed by labeling with a Janelia Fluor 635i HaloTag Ligand (kindly provided by the Lavis laboratory, Janelia HHMI). JF635i was stored as a 1 mM stock solution in dimethyl sulfoxide and diluted to 1:3000 within 20 min of labeling cells. Cells were incubated at 37 °C for 30 min with a 1:3000 dilution of the JF635i dye in Dulbecco's modified Eagle's medium (without fetal bovine serum and Pen/Strep) to a working concentration of 0.33 μM. Cells were then washed three times with 1X PBS and fixed at room temperature for 15 min with 4% formaldehyde (diluted from 16% formaldehyde (w/v) solution; Pierce, 28908). Fixed cells were labeled with DAPI for 5 min at room temperature using NucBlue ReadyProbes Reagent (Thermo Fisher Scientific) and then washed three times and kept in 200 μl 1X PBS for visualization. Images were acquired with a 10X magnification Plan Apo Lambda objective in a 2 × 2 tile matrix of each well using the ImageXpress Micro Confocal High Content Imaging System (Molecular Devices), paired with MetaXpress 6 software. Images at four spectral wavelengths (Cy5: [ex: 649 nm, em: 667 nm]; Cy3: [ex: 554 nm, em: 568 nm]; FITC: [ex: 495 nm, em: 519 nm]; DAPI: [ex: 350 nm, em: 465 nm]) were acquired based on the respective fluorophore (JF635i: [ex 635 nm, em: 657 nm]; dsRed: [ex: 546 nm, em: 574 nm]; EGFP: [ex: 489 nm, em: 509 nm]; DAPI: [ex: 350 nm, em: 465 nm]) present during experimentation. A Z-stacked image was rendered across three focal planes to generate a “Best Focus” reconstruction of cells at each wavelength. For analysis, successful transfections were identified by selecting cells exhibiting a minimum Cy3 (dsRed) threshold of 100,000 integrated fluorescence intensity.

### Data analysis

For all analyzed data from the immunoblots, band intensity was first normalized to their respective β-actin loading control in each lane. Data displayed as scatter plots or bar graphs are expressed as mean ± SEM. Box plots depict the median, 25th and 75th percentile (box), and 10th and 90th percentile (whiskers). Conductance-voltage relationships were fitted using a form of the Boltzmann equation (Equation 1), where G is the normalized conductance, V is the voltage applied, V_1/2_ is the half-activation voltage, and k is a fitted value reflecting the steepness of the curve.

(1) $$G\left(V\right)=1/\;\left(1+e_{1/2}^{-\left(V-V\right)/k}\right)$$

Conductance-voltage relationships were fit for each individual cell, and the extracted fit parameters were used for statistical calculations.

The voltage dependence of Kv2.1 inactivation was determined by a three-pulse protocol. The channels were held at a holding potential of −100 mV, then a depolarization to +60 mV for 200 ms (I_1_), followed by a brief −100 mV, then stepping from −100 mV to +80 mV in 20 mV increments for 4 s for channel inactivation and followed by another test pulse to +60 mV for 200 ms (I_2_) to determine the fraction of inactivated channel. The normalized current was calculated dividing the I_2_/I_1_ currents and was plotted against the individual step voltages. As Kv2.1:Kv6.1 coexpression leads to prominent U-shaped inactivation curves, the voltage dependence of inactivation was only fit in the range of voltages preceding the upturn of the relationship. These segments were fit with a Boltzmann-type function (Equation 2), where V is the voltage applied, V_1/2_ is the half-inactivation voltage, k is a fitted value reflecting the steepness of the curve, and C is a constant that reflects the residual current at maximal inactivation (*i.e.* the level of the “trough” of the inactivation-voltage relationship).

(2) $$G\left(V\right)=C+\left(1-C\right)/\left(1+e_{1/2}^{\left(V-V\right)/k}\right)$$

Statistical tests are described in each Figure legend, along with the resulting *p* value. All data were analyzed using SigmaPlot or Origin Pro software (https://www.originlab.com/). For all tests, *p* < 0.05 was considered significant.

### Collection of data from human subjects

Sequence data arising from human samples was collected under REB18-1486 (Care4Rare Canada SOLVE, University of Calgary) and subjects provided consent for investigation. Work with human patients abides by the Declaration of Helsinki principles.

## Data availability

All original data is available upon request by contacting the corresponding author.

## Conflict of interest

The authors declare that they have no conflicts of interest with the contents of this article.
